# Effects on mechanical power of different devices used for inhaled sedation in a bench model of protective ventilation in ICU

**DOI:** 10.1186/s13613-024-01245-x

**Published:** 2024-01-29

**Authors:** Pierre-Louis Pellet, Neven Stevic, Florian Degivry, Bruno Louis, Laurent Argaud, Claude Guérin, Martin Cour

**Affiliations:** 1 Hospices Civils de Lyon, Service de Médecine Intensive -Réanimation, Hôpital Edouard Herriot, 5 Place d’Arsonval, 69437 Lyon Cedex 03, France; 2 Université de Lyon, Université Claude Bernard Lyon 1, Faculté de Médecine Lyon-Est, 69373 Lyon, France; 3 Institut Mondor de Recherches Biomédicales INSERM 955 CNRS 7000, Créteil, France

**Keywords:** Sevoflurane, MIRUS, AnaConDa, Dead space, Acute respiratory distress syndrome, Sedation, Mechanical power, Costa index

## Abstract

**Background:**

Inhaled sedation during invasive mechanical ventilation in patients with acute respiratory distress syndrome (ARDS) has received increasing attention. However, inhaled sedation devices increase dead-space ventilation and an undesirable effect is the increase in minute ventilation needed to maintain CO_2_ removal. A consequence of raising minute ventilation is an increase in mechanical power (MP) that can promote lung injury. However, the effect of inhaled sedation devices on MP remains unknown.

**Methods:**

We conducted a bench study to assess and compare the effects of three devices delivering inhaled sevoflurane currently available in ICU (AnaConDa-50 mL (ANA-50), AnaConDa-100 mL (ANA-100), and MIRUS) on MP by using a test lung model set with three compliances (20, 40, and 60 mL/cmH_2_O). We simulated lung-protective ventilation using a low tidal volume and two levels of positive end-expiratory pressure (5 and 15 cmH_2_O) under ambient temperature and dry conditions. Following the insertion of the devices, either the respiratory rate or tidal volume was increased in 15%-steps until end-tidal CO_2_ (EtCO_2_) returned to the baseline value. MP was calculated at baseline and after EtCO_2_ correction using a simplified equation.

**Results:**

Following device insertion, the EtCO_2_ increase was significantly greater with MIRUS (+ 78 ± 13%) and ANA-100 (+ 100 ± 11%) than with ANA-50 (+ 49 ± 7%). After normalizing EtCO_2_ by adjusting minute ventilation, MP significantly increased by more than 50% with all inhaled sedation devices compared to controls. The lowest increase in MP was observed with ANA-50 (*p* < 0.05 versus ANA-100 and MIRUS). The Costa index, another parameter assessing the mechanical energy delivered to the lungs, calculated as driving pressure × 4 + respiratory rate, significantly increased by more than 20% in all experimental conditions. Additional experiments performed under body temperature, ambient pressure, and gas saturated with water vapor conditions, confirmed the main results with an increase in MP > 50% with all devices after normalizing EtCO_2_ by adjusting minute ventilation.

**Conclusion:**

Inhaled sedation devices substantially increased MP in this bench model of protective ventilation, which might limit their benefits in ARDS.

## Background

Stemming from the landmark ARMA trial, the primary goal of protective invasive mechanical ventilation (IMV) in acute respriatory distress syndrome (ARDS) is to limit excessive stress and strain applied to the lung [1–3]. Therefore, setting tidal volume (*V*_T_) between 4 and 8 mL/kg predicted body weight and maintaining plateau pressure below 30 cm H_2_O are strongly recommended [2, 3]. Owing to the combination of low V_T_ and increased physiological dead-space (a hallmark of ARDS), decarboxylation is often impaired in ARDS [4]. According to a recent meta-analysis, patients with ARDS who experience hypercapnia resulting from factors other than protective ventilation aimed at reducing lung stress or strain may have a higher risk of mortality [4]. The main way to limit hypercapnia without increasing minute ventilation is to reduce the instrumental dead-space. For this reason, in ARDS, a heated humidifier is preferred over a heat and moisture exchanger as it does not increase the instrumental dead space, while ensuring the mandatory humidification and heating of inspired gases [3, 5].

ARDS patients under IMV commonly require analgesia and sedation in the early stage of management [6]. Inhaled sedation with halogenated anesthetics is an emerging alternative to usual intravenous sedation in intensive care units (ICU) [7–10], including in ARDS patients in whom it may improve oxygenation [11, 12]. It may shorten weaning from IMV and reduce opioid consumption compared to intravenous drugs, without safety concerns. Moreover, halogenated anesthetics have anti-inflammatory properties, which could be beneficial in ARDS [11, 13, 14].

Two types of devices are currently available for inhaled sedation in the ICU: the anesthetic-conserving device AnaConDa (Sedana Medical, Uppsala, Sweden) using syringe pumps and a vaporizer filter, and the MIRUS device (TIM GmBH, Koblenz, Germany) using an electronic gas delivery system with a reflective filter [8, 15, 16]. Because both devices are placed between the Y-piece and the patient, the instrumental dead-space increases with the volume of the filter [8]. The risk of hypercapnia is further enhanced by the reflection of CO_2_ in the devices leading to mandatory CO_2_ rebreathing during inspiration [8, 17].

Increasing minute ventilation (through an increase in respiratory rate [RR] and/or *V*_T_) may be used to dampen the device-induced PaCO_2_ rise. However, it inevitably increases the mechanical energy applied to the lungs, which can be estimated by mechanical power (MP). MP is a calculation that integrates strain (*V*_T_), stress (pressure), and the rate of lung deformation [18]. The increase in MP increases the risk of ventilator-induced lung injury and poor patient outcome [19]. This could counteract the beneficial effects of inhaled sedation during protective IMV for ARDS.

In this bench study of lung-protective ventilation, we assessed and compared the effects of three inhaled sedation devices on MP when end-tidal CO_2_ was kept constant by adjusting the minute ventilation.

## Methods

We tested, on the bench, the effects of the inhaled sedation devices currently used in ICU on MP in a test lung model set with low compliance. We aimed to simulate lung-protective ventilation (6 mL/kg predicted body weight) in an adult patient with low lung compliance, as observed in ARDS. The experiment was performed in a dedicated room in the medical ICU of Edouard Herriot University Hospital in Lyon, France. Due to its in vitro nature, no agreement with an ethical committee was required for this study. Inhaled sedation devices were provided by the corresponding manufacturers, and halogenated anesthetic gas was provided by the pharmacist of the hospital.

### Setup

**Fig. 1:**
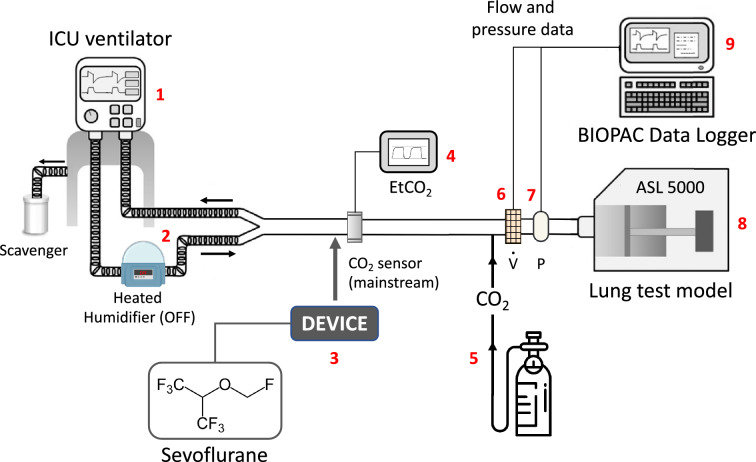
Bench model (ambient temperature pressure dry conditions): (1) intensive care unit (ICU) ventilator, (2) heated humidifier placed on inspiratory limb and switch-off, (3) inhaled sedation device, (4) CO_2_ sensor and monitor, (5) CO_2_ delivery system, (6) post-device airflow transducer ($\dot{V}$), (7) post-device airway pressure transducer (P), (8) ASL 5000 lung test, (9) BIOPAC data logger

The main experimental setup (bench model #1, Fig. 1) was conducted under ATPD (ambient temperature pressure dry) conditions and consisted of the following components: an ICU ventilator (Evita 4, Dräger Medical, Germany) set in volume control mode with a squared inspiratory flow, a heated humidifier (MR850, Fisher & Paykel Healthcare, New Zealand) placed on the inspiratory limb of the ventilator circuit (switched-off), and an anesthetic gas-scavenging system connected to the expiratory valve (FlurAbsorb, Sedana Medical). The ventilator was connected to a lung model (ASL 5000, Ingmar Medical Inc., Pittsburgh, PA, USA) set in the passive condition, with a single fixed resistance of 5 cm H_2_O/L/s in both the inspiratory and expiratory directions. Lung compliance was set at 60, 40, or 20 mL/cm H_2_O. The Y-piece of the double-limb ventilator tubing was connected to the lung test via a 120 mL dead-space circuit (i.e., close to the anatomical dead-space of an intubated patient) [21]. A CO_2_ bottle was connected to the lung test inlet to deliver a continuous CO_2_ flow adjusted (with a rotameter) to achieve a stable end-tidal CO_2_ of 40 mmHg at baseline. Three devices for inhaled sedation were successively added to the circuit: AnaConDa-50 mL (ANA-50), AnaConDa-100 mL (ANA-100) (Sedana Medical), and MIRUS (TIM GmBH). Sevoflurane was used as the halogenated anesthetic gas with an expired fraction (FeSevo) of 1.3%. The internal volume of the device, that is, the additional instrumental dead space, was estimated to be approximately 50 mL for ANA-50, and 100 mL for ANA-100 and MIRUS [8, 15, 16].

**Fig. 2:**
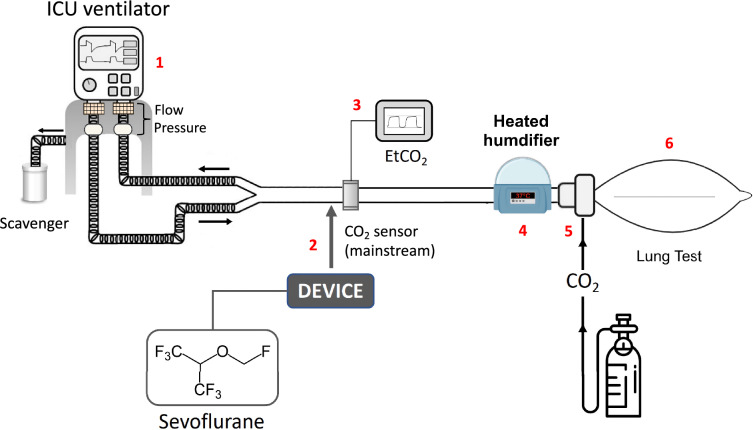
Bench model (body temperature pression saturated conditions): (1) intensive care unit (ICU) ventilator, (2) inhaled sedation device, (3) CO_2_ sensor and monitor, (4) heated humidifier set at 37 °C, (5) CO_2_ delivery system, (6) lung test

To make our assessment closer to the clinical practice of protective IMV and to avoid potential experimental biases (e.g., sub-optimal performances of devices under dry conditions at room temperature), we carried out an additional procedure (bench model #2, Fig. 2) under body temperature (37 °C), ambient pressure, and gas saturated with water vapor (BTPS) conditions. To prevent any damage in the ASL 5000 lung model due to humidified air, a Maquet 1 L test lung (Getinge, Solna, Sweden) with 25 mL/cmH_2_O compliance and 15 cmH_2_O/L/s resistance was used, and the volumetric dead space of the circuit without any inhaled sedation device was 150 mL.

### Experimental protocol

The ventilator was set with 400 mL *V*_T_, 60 L/min inspiratory flow, 0.4 s end inspiratory occlusion time, 20 breaths/min RR, and inspired oxygen fraction 21%. The PEEP was set at either 5 or 15 cmH_2_O.

First, the ventilator was running through the circuit detailed in Fig. 1 (bench model #1) without inhaled sedation device, until EtCO_2_ was stable at 40 mmHg. This defined the baseline control (CTRL). Subsequently, one of the three inhaled sedation devices was added, with an expected increase in EtCO_2_. The baseline for the inhaled sedation device was obtained when FeSevo reached 1.3% and EtCO_2_ remained stable. EtCO_2_ was then returned to 40 ± 2 mmHg using two different intervention strategies. In two separate runs, either *V*_T_ or RR was increased in 15%-steps, namely, 60 mL and 3 breaths/min, respectively, every 2.5 min until 40 ± 2 mmHg EtCO_2_ was resumed. At that time, a 3-s inspiratory pause and a 3-s expiratory pause were performed to measure the plateau pressure and total PEEP, respectively.

In the bench model #2 (BTPS conditions), the procedure described above was replicated at the two PEEP levels; however, a single compliance of 25 mL/cm H_2_O was tested.

### Outcomes

The primary outcome was MP computed from the simplified equation and expressed in J/min: MP = 0.098 × RR × *V*_T_ × [peak pressure − (0.5 × driving pressure)] where the driving pressure was calculated as the difference between the plateau pressure and total PEEP [18–20]. During protective ventilation for ARDS, MP is estimated to be 10–20 J/min and values greater than 12 J/min have been shown to be associated with increased risk of mortality [20]. As flow and pressure transducers were placed after inhaled sedation devices, MP calculation did not include resistive pressures related to the devices.

Secondary outcomes included the driving pressure and the Costa index, which are two variables that estimate lung stress and/or strain independently of resistive pressures (unlike MP), and that are associated with poor prognosis in ARDS [18–20, 22]. The Costa index was calculated as follows: 4 × driving pressure + RR [22]. Device-induced increase in EtCO_2_ was also analyzed.

### Data analysis

Before the experiment, the ventilator was fully checked, and the airway pressure (Paw) transducers and pneumotachographs were calibrated using a manometer (717 1G, Fluke Biomedical, Everett, Washington, USA), and a 1 ± 0.012 L calibration pump (Viasys, Hochberg, Germany), respectively, at room temperature. The CO_2_ measurement device was calibrated according to the manufacturer instructions. Airflow ($\dot{V})$ and Paw were measured after the inhaled sedation device (i.e., at the ASL 5000 inlet) by using a pneumotachograph (3700 series, Hans Rudolph, Shawnee, Kansas, USA) and a pressure transducer (Gabarith PMSET 1DT-XX, Becton Dickinson, Singapore), respectively (Fig. 1). $\dot{V}$ and Paw signals were sent to a datalogger (MP150, Biopac Systems Inc., Goletta, CA, USA), sampled at 200 Hz, and stored for further analysis. CO_2_ was measured using a mainstream sensor (Dräger Medical, Germany) and EtCO_2_ was monitored using a built-in ventilator device (Fig. 1). Sevoflurane concentration was monitored using a dedicated device for each inhaled sedation device brand.

The respiratory variables recorded in the data logger were automatically measured offline using an application developed in MATLAB (R2021b, MathWorks). The variables required for the calculation of MP were obtained from at least 6 breaths. When indicated, *V*_T_ was corrected for sevoflurane density (corrected *V*_T_ = measured V_T_/0.993) at a FeSevo 1.3% because flow sensors were calibrated with air [23]. No correction for CO_2_ was performed.

For complementary experiments under BTPS conditions, respiratory data were obtained from ventilator transducers (a single measure was performed for each and there was no V_T_ correction for sevoflurane).

### Statistical analysis

Data are expressed as means ± standard deviation (SD). Continuous data including MP were compared among the four experimental groups (CTRL, MIRUS, ANA-100, ANA-50) for each of the experimental conditions (i.e., 3 compliances × 2 levels of PEEP × 3 states (baseline, *V*_T,_ and RR correction)) using one-way analysis of variance (ANOVA) and Tukey’s test for multiple pairwise comparisons or by the Kruskal–Wallis test and Dunn’s test for multiple comparison, as appropriate. For confirmatory experiments under BTPS conditions, no statistical analysis was performed for MP because a single measure was recorded for each condition. For EtCO_2_, means were compared using ANOVA (several values were obtained for each device). Statistical analyses were performed using GraphPad Prism 9 software (GraphPad Software, La Jolla, CA, USA). Statistical significance was defined as a value of *p* < 0.05.

## Results

**Fig. 3:**
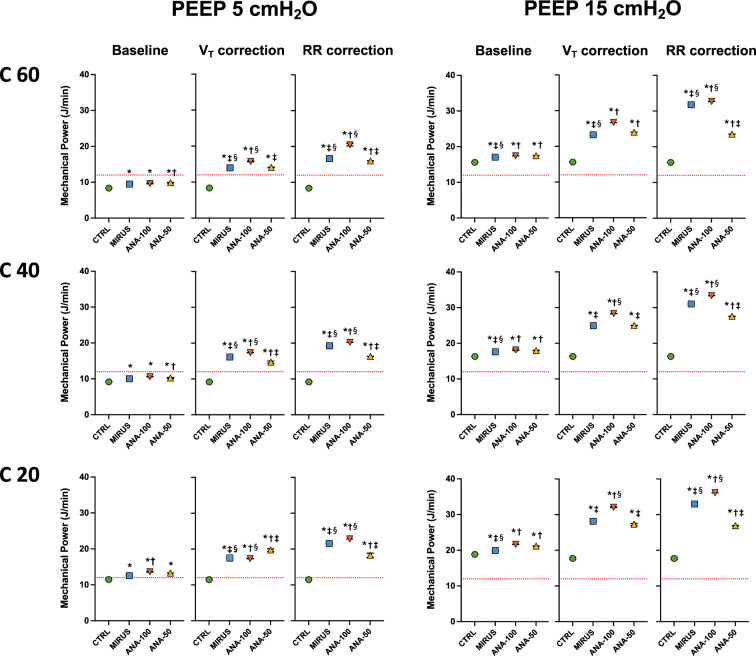
Effect of inhaled sedation devices on mechanical power under ambient temperature pressure dry conditions. The dashed red lines represent the value of mechanical power (12 J/min) above which there is a risk of excess mortality. Error bars indicate standard deviation. C: compliance of the lung test (expressed in mL/cmH_2_O); CTRL: Control group (green circle, no device); MIRUS: Mirus^™^ device (blue square); ANA-100: AnaConDa-100 mL device (orange triangle); ANA-50: AnaConDa-50 mL device (yellow triangle). ^a^*p* < 0.05 vs. CTRL †*p* < 0.05 vs. MIRUS ‡*p* < 0.05 vs. ANA-100 §*p* < 0.05 vs. ANA-50

**Fig. 4:**
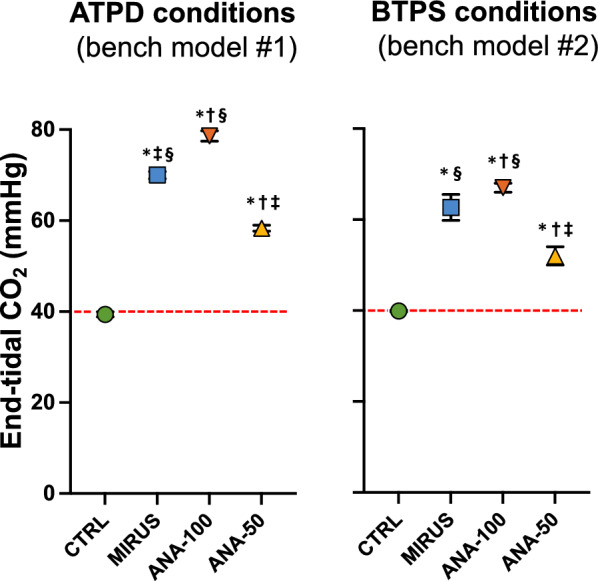
Effect of inhaled sedation devices on end-tidal CO_2_. The effects of inhaled sedation devices on end-tidal CO_2_ were first analyzed under ambient temperature pressure dry (ATPD) conditions (bench model #1) and then under body temperature pression saturated (BTPS) conditions (humid conditions, bench model #2). Experiments in ATPD and BTPS conditions were independent Dashed redlines indicate the baseline value of end-tidal CO_2_ (40 mmHg). Error bars indicate standard deviation. CTRL: Control group (green circle, no device); MIRUS: Mirus™ device (blue square); ANA-100: AnaConDa-100 mL device (orange triangle); ANA-50: AnaConDa-50 mL device (yellow triangle). *p < 0.05 vs CTRL, †*p* < 0.05 vs MIRUS, ‡*p* < 0.05 vs ANA-100, §*p* < 0.05 vs ANA-50

In the bench model under ATPD conditions (Fig. 1), at baseline, for each PEEP level and compliance, MP was slightly but significantly (*p* < 0.05) higher with inhaled sedation devices than in CTRL (Fig. 3). The use of any inhaled sedation device resulted in a significant increase in EtCO_2_ compared with CTRL (Fig. 4). The magnitude of the increase in EtCO_2_ was significantly (*p* < 0.05) greater with MIRUS (+ 78 ± 13%) and ANA-100 (+ 100 ± 11%) than with ANA-50 (+ 49 ± 7%); the highest increase in EtCO_2_ was observed with ANA-100 (Fig. 4).

Ventilatory data, including peak pressure, plateau pressure, total PEEP, V_T_, and RR at baseline and after EtCO_2_ correction, through increases in either *V*_T_ or RR, are reported in Table 1 (for all compliances and PEEP levels). At a PEEP of 5 cm H_2_O, MP increased by > 50% after EtCO_2_ correction by increasing RR or V_T_ with all devices (Fig. 3). Similarly, at a PEEP of 15 cmH_2_O, MP increased by more than 50% for all devices after V_T_ or RR correction (Fig. 3). The increase in MP after EtCO_2_ correction was significantly lower with ANA-50 under almost all conditions compared with the two devices with higher volumetric dead space (Fig. 4). Conversely, ANA-100 resulted in a significantly higher increase in MP in almost all conditions compared with the other devices. Part of the increase in MP following *V*_T_ or RR correction was due to an increase (up to 4 cm H_2_O) in the total PEEP (Table 1).

**Table 1:** Effect of inhaled sedation devices on ventilatory pressures, driving pressure, and Costa index at lung compliances of 60, 40 and 20 mL/cmH_2_O

	PEEP 5 cmH_2_O				PEEP 15 cm H_2_O			
	Compliance 60 mL/cm H_2_O							
Baseline	CTRL	MIRUS	ANA-100	ANA-50	CTRL	MIRUS	ANA-100	ANA-50
Ppeak	**16**	16	16	16	**26**	26^a^	27^a^	27^a^
Pplat	**11**	12^a^	12^a^	12^a^	**21**	22^a^	23^a^	23^a^
PEEPt	**5**	6^a^	6^a^	6^a^	**15**	16^a^	16^a^	17^a^
ΔP	**6**	6^a^	6^a^	6^a^	**6**	6^a^	6^a^	6^a^
4×ΔP + RR	**44**	45^ad^	45^a^	45^ab^	**44**	45^ad^	45^a^	45^ab^
*V*_T_ correction								
Ppeak	**16**	18^a^	19^a^	19^a^	**26**	29^a^	30^a^	29
Pplat	**11**	15^a^	16^a^	15^a^	**21**	25^a^	26^a^	25^a^
PEEPt	**5**	6^a^	6^a^	6^a^	**15**	16^a^	17^a^	17^a^
ΔP	**6**	9^a^	9^a^	8^a^	**6**	8^a^	9^a^	8^a^
Final *V*_T_	**400**	560	580	520	**400**	580	580	520
4 × ΔP + RR	**44**	55^acd^	57^abd^	53^abc^	**44**	54^acd^	57^abd^	53^abc^
RR correction								
Ppeak	**16**	19^a^	20^a^	19^a^	**26**	30^a^	30^a^	28^a^
Pplat	**11**	15^a^	16^a^	14^a^	**21**	26^a^	26^a^	24^a^
PEEPt	**5**	8^a^	8^a^	7^a^	**15**	19^a^	19^a^	17^a^
ΔP	**6**	7^a^	8^a^	7^a^	**6**	7^a^	7^a^	7^a^
Final RR	**20**	32	35	29	**20**	35	35	26
4 × ΔP + RR	**44**	61^a^	67^a^	57^a^	**44**	65^a^	67^a^	57^a^

	Compliance 40 mL/cmH_2_O							
Baseline	CTRL	MIRUS	ANA-100	ANA-50	CTRL	MIRUS	ANA-100	ANA-50
Ppeak	**18**	18	19	19	**29**	29	29	29
Pplat	**14**	15^a^	15^a^	15^a^	**24**	25^a^	25^a^	25^a^
PEEPt	**5**	6^a^	6^a^	6^a^	**15**	16^a^	16^a^	16^a^
ΔP	**9**	9^a^	9^a^	9^a^	**9**	9^a^	9^a^	9^a^
4 × ΔP + RR	**55**	56^a^^cd^	56^a^^bd^	57^a^^bc^	**55**	56^a^^cd^	56^a^^bd^	57^a^^bc^
*V*_T_ correction								
Ppeak	**18**	22^a^	23^a^	22^a^	**29**	32^a^	33^a^	32^a^
Pplat	**14**	19^a^	20^a^	18^a^	**24**	28^a^	30^a^	28^a^
PEEPt	**5**	6^a^	6^a^	6^a^	**15**	16^a^	17^a^	16^a^
ΔP	**9**	13^a^	14^a^	12^a^	**9**	12^a^	13^a^	12^a^
Fin^a^l V_T_	**400**	580	580	520	**400**	580	580	520
4xΔP + RR	**55**	71^a^^cd^	74^a^^bd^	68^a^^bc^	**55**	71^a^^cd^	74^a^^bd^	68^a^^bc^
RR correction								
Ppeak	**18**	21^a^	21^a^	20^a^	**29**	32^a^	32^a^	31^a^
Pplat	**14**	18^a^	17^a^	17^a^	**24**	28^a^	28^a^	27^a^
PEEPt	**5**	8^a^	7^a^	7^a^	**15**	18^a^	18^a^	17^a^
ΔP	**9**	10^a^	10a	10^a^	**9**	10^a^	10^a^	10^a^
Final RR	**20**	35	35	29	**20**	35	35	29
4xΔP + RR	**55**	75^acd^	76^abd^	68^abc^	**55**	75^acd^	76^abd^	68^abc^

	Compliance 20 mL/cm H_2_O							
Baseline	CTRL	MIRUS	ANA-100	ANA-50	CTRL	MIRUS	ANA-100	ANA-50
Ppeak	**26**	25	26	26	**36**	36	37^a^	37
Pplat	**22**	23^a^	23^a^	23^a^	**32**	33^a^	34^a^	34^a^
PEEPt	**5**	5^a^	6a	6^a^	**15**	16^a^	16^a^	16^a^
ΔP	**17**	17^a^	17a	17a	**17**	17^a^	17^a^	17^a^
4 × ΔP + RR	**88**	88^cd^	90^ab^	90^ab^	**88**	88^cd^	90^ab^	90^ab^
*V*_T_ correction								
Ppeak	**26**	31^a^	34a	32^a^	**36**	41^a^	43^a^	41^a^
Pplat	**22**	28^a^	32^a^	29^a^	**32**	38^a^	41^a^	38^a^
PEEPt	**5**	5^a^	6^a^	6^a^	**15**	16^a^	16^a^	16^a^
ΔP	**17**	22^a^	25^a^	23^a^	**17**	22^a^	24^a^	22^a^
Final *V*_T_	**400**	580	580	520	**400**	580	580	520
4xΔP + RR	**88**	109^acd^	123^abd^	114^abc^	**88**	109^acd^	123^abd^	114^abc^
RR correction								
Ppeak	**26**	27^a^	27^a^	27^a^	**36**	37^a^	38a	37^a^
Pplat	**22**	24a	24^a^	24^a^	**32**	34a	35^a^	34^a^
PEEPt	**5**	6^a^	6^a^	6^a^	**15**	17^a^	17^a^	16^a^
ΔP	**17**	17^a^	18^a^	18^a^	**17**	18^a^	18^a^	18^a^
Final RR	**20**	35	35	29	**20**	35	35	26
4 × ΔP + RR	**88**	106^acd^	107^abd^	102^abc^	**88**	106^acd^	107^abd^	102^abc^

Data are expressed as mean and standard deviations are not shown because they are equal to 0 for each parameter

*CTRL* Control group (no device, values reported in bold), *MIRUS* Mirus device, *ANA-100* AnaConDa device with 100 mL volumetric dead space; ANA-50: AnaConDa device with 50 mL volumetric dead space; PPeak: peak pressure (cmH_2_O); PPlat: plateau pressure (cmH_2_O); PEEPt: total positive end-expiratory pressure (cmH_2_O); ΔP: driving pressure (cmH_2_O); 4×ΔP + RR: Costa index (arbitrary units)

Final *V*_T_: tidal volume (expressed in mL) set on the ventilator to maintain end-tidal CO_2_ at 40 ± 2 mmHg; Final RR: respiratory rate (breaths/minute) set on the ventilator to maintain end-tidal CO_2_ at 40 ± 2 mmHg

^a^*p* < 0.01 vs. CTRL

^b^*p* < 0.01 vs. MIRUS

^c^*p* < 0.01 vs. ANA-100

^d^*p* < 0.01 vs. ANA-50

As shown in Table 1, the use of all inhaled sedation devices led to a significant increase (from 20 to 38%) in both driving pressure and Costa index after EtCO_2_ correction compared with CTRL. Under most conditions, the highest increase in the Costa index was observed with ANA-100 and the lowest with ANA-50 (Table 1).

**Fig. 5:**
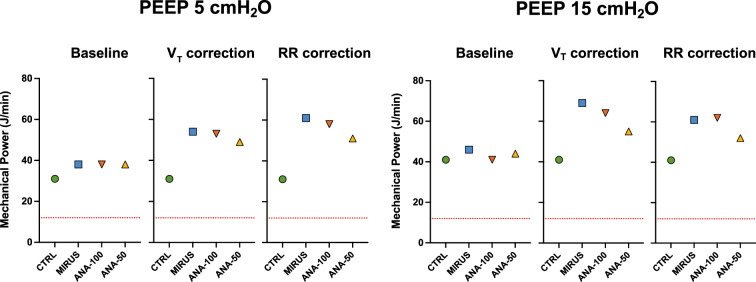
Effect of inhaled sedation devices on mechanical power under body temperature pressure saturated conditions. Dashed red lines represent the mechanical power value (12 J/min) above which there is a risk of excess mortality. CTRL: Control group (green circle, no device); MIRUS: Mirus^™^ device (blue square); ANA-100: AnaConDa-100 mL device (orange triangle); ANA-50: AnaConDa-50 mL device (yellow triangle)

In the additional model set up in BTPS conditions (Fig. 2), the results mostly mirrored those obtained from the main model under ATPD conditions, with both substantial increases in EtCO_2_ and in MP (> 50%) with all inhaled sedation devices compared to CTRL (Figs. 4, 5). As in dry conditions, the increase in both EtCO_2_ and MP was higher for devices with a large geometric dead space (i.e., MIRUS and ANA-100) than for those with a smaller dead space (i.e., ANA-50) (Figs. 4, 5). The increase in EtCO_2_ was significantly (*p* < 0.01) lower under BTPS conditions than under dry conditions for all the devices. At low PEEP, the Costa index increased by less than 20% after EtCO_2_ correction for all devices and all compliances. At high PEEP, the Costa index increased from 66 points at baseline to 143, 124, and 99 points after EtCO_2_ correction through an increase in RR with MIRUS, ANA-100, and ANA-50, respectively; it increased to 158, 175, and 130 points after EtCO_2_ correction through an increase in V_T_ with MIRUS, ANA-100, and ANA-50 device, respectively.

## Discussion

The main findings of this bench study are as follows: (1) inhaled sedation devices significantly increased MP, often well above 50%, due to the increase in minute ventilation required to maintain EtCO_2_ at baseline values, and (2) inhaled sedation devices were not equivalent in terms of changes in MP in our model of lung-protective ventilation.

To our knowledge, only one previous bench study has assessed an inhaled sedation device in the setting of lung-protective ventilation in ICU with a *V*_T_ of 6 mL/kg [24]. The authors, using ANA-100 with FeSevo set at 0.8%, concluded that it was not possible to maintain baseline EtCO_2_ with such a low *V*_T_ without increasing the RR above 40 cycles/min. In our bench study, we did not confirm this finding because EtCO_2_ could be restored to baseline values after increasing RR to a maximum of 35 cycles/min following the use of inhaled sedation devices, for all experimental conditions [24]. Conversely, in our bench model, at constant RR, it was not possible to maintain normocapnia when using the devices with 100 mL volumetric dead space (i.e., ANA-100 and MIRUS) without increasing *V*_T_ above 8 mL/kg predicted body weight. The discrepancy between the previous and present study may be related to the impact of the inhaled sedation devices on EtCO_2_, which strongly depends on the percentage fraction of dead space to V_T_ and, hence, on the experimental conditions. In our model, we chose a circuit with a dead space of about 2 mL/kg to approximate the anatomical dead space of an intubated patient [21]. This may differ from ARDS patients in whom the dead space can be higher, increasing the risk of hypercapnia [25].

Under our experimental conditions simulating lung-protective ventilation in ARDS, we found that an inhaled sedation device with the lowest dead space still led to a significant and meaningful increase in both EtCO_2_ and MP. This result has not been confirmed by a recent clinical study in non-ARDS ICU patients receiving standard ventilation (*V*_T_ 8–10 mL/kg), which compared ANA-50 and ANA-100 with conventional intravenous sedation [26]. Indeed, the authors only found an increase in PaCO_2_ or in minute ventilation with ANA-100. Nevertheless, in the control group of this study, gas humidification was provided by a heat and moisture exchanger, whose dead space was far from negligible (35–50 mL). This may explain why there was no difference between the ANA-50 and the control group. However, the findings in patients without ARDS should not be extrapolated to those with ARDS in whom heated humidifiers are preferred over heat and moisture exchangers.

In our bench model, we maintained EtCO_2_ at a baseline value of 40 mmHg by increasing RR or *V*_T_. In clinical practice, hypercapnia can be tolerated in ARDS, particularly when the purpose is to limit lung stress or strain [4]. This may facilitate the use of inhaled sedation in ARDS patients. A randomized trial showed that protective ventilation (*V*_T_ 6–8 mL/kg) using ANA-100 with sevoflurane was feasible in patients with ARDS and might even improve oxygenation [11]. Nevertheless, one day after inclusion, although not statistically different, PaCO_2_, V_T_, RR, airway resistance, and plateau pressure were higher in patients sedated with ANA-100 than in those receiving intravenous sedation [11]. Therefore, it is likely that the use of an inhaled sedation device increased MP compared with controls because its calculation includes *V*_T_, RR, and peak pressure [18]. Another randomized trial involving 60 patients who underwent protective ventilation with lower *V*_T_ (4–6 mL/kg and > 350 ml), mostly for ARDS, it was demonstrated that sedation with ANA-100 was feasible compared to intravenous sedation with propofol [27]. However, it was observed that minute ventilation was markedly higher (up to 50%) in the ANA-100 group despite lower pH, which likely resulted in a substantial increase in lung stress and MP [27]. Whether inhaled sedation devices with lower dead space (e.g., ANA-50) allow lung-protective ventilation in patients with ARDS and improve long-term outcomes is under investigation [28]. It would be interesting to assess the effects of sedation devices on MP or other variables such as the Costa index in ongoing or future trials.

The main finding of the present study was that the use of all inhaled sedation devices tested in our experimental setup resulted in a marked increase in mechanical power (> 50%) and, to a lesser extent, in the Costa index, which also estimates the mechanical stress imposed on the lungs, excluding that related to resistive pressures. The results under humid conditions were consistent with those under dry conditions, even though the values of MP were higher under humid conditions because of the higher peak pressure owing to the higher flow-resistance of the test lung (15 versus 5 cm H_2_O/L/s). Importantly, the devices had different effects on MP or other estimates of lung stress. ANA-50 limited the increase in MP compared with ANA-100 and MIRUS. Moreover, despite the similar internal dead space (100 mL), the increase in CO_2_, and consequently in MP (after EtCO_2_ correction), was higher in ANA-100 than in MIRUS, especially at low compliance and high PEEP. This suggests that the reflection of CO_2_ was higher in ANA-100 and depended on the inspiratory pressure. As MP is associated with outcomes in ARDS [18, 19], inhaled sedation devices with low dead space should theoretically be preferred for lung-protective ventilation in ARDS patients, even if clinical data supporting this statement are lacking. Notably, to avoid an increase in dead space in patients with ARDS, inhaled sedation devices can be placed on the inspiratory branch (before the Y-piece) [29, 30]. In this case, there is no reflection (instead of > 90%) of halogenated gas, leading to a major increase in gas consumption, and, in turn, in economic costs and environmental impact [31].

Our study had several strengths and limitations. First, we compared the three inhaled sedation devices currently available in the market using the same bench model of lung-protective ventilation with several conditions of PEEP and compliances. Such studies cannot be conducted in humans. Second, we chose as primary outcome MP, a variable that estimates, based on thermodynamic principles, the amount of energy that is delivered to the lung, rather than dead space or CO_2_ as in most studies [18]. Although imperfect, the MP summarizes the contributions of static and dynamic ventilatory parameters that may participate in ventilator-induced lung injury [32]. Moreover, our MP results were confirmed in the analysis using driving pressure and the Costa index, two other validated parameters that estimate lung stress but do not consider the dynamic component (i.e., resistive pressure) [18–20, 22]. The relationship between the expired sevoflurane fraction and CO_2_ reflection is such that an increased sevoflurane fraction is associated with a lower CO_2_ reflection [17, 24]. We chose a higher FeSevo than that used in a clinical trial for ARDS patients (1.3% versus 0.6–0.8%) [11]. Consequently, we may have underestimated the potential CO_2_ reflection that could be observed when using a lower sevoflurane fraction, which is common in ICU. Thus, selecting a high FeSevo could limit the clinical relevance of our findings. The main limitation of our study was the use of a bench model that did not simulate the effects of halogenated anesthetics on lung mechanics and gas exchange, which may also vary depending on the anesthetic gas (sevoflurane, isoflurane, desflurane, etc.). Additionally, in patients with ARDS, the distribution of mechanical power within the lungs plays a crucial role in determining regional ventilator-induced lung injury [32]. Unfortunately, our model did not account for this aspect. Therefore, our results cannot be generalized to clinical practice.

## Conclusions

In this bench study of protective ventilation for ARDS in the ICU, we observed that using inhaled sedation devices led to a substantial increase in the mechanical energy applied in the lung test, as measured by either the MP or Costa index, when EtCO_2_ was maintained at baseline levels. The device with the lowest dead space, ANA-50, had the least impact on both the MP and Costa index.

## Data Availability

The dataset used and/or analyzed during the current study are available from the corresponding author on reasonable request.

## Abbreviations

ANA-50: AnaConDa device with 50 mL dead space
ANA-100: AnaConDa device with 100 mL dead space
ANOVA: One-way analysis of variance
ARDS: Acute respiratory distress syndrome
ATPD: Ambient temperature, ambient pressure, dry gas
BTPS: Body temperature, ambient pressure, gas saturated with water vapor
CTRL: Control group
EtCO_2_: End-tidal CO_2_
FeSevo: Expired fraction of sevoflurane
ICU: Intensive care unit
IMC: Invasive mechanical ventilation
MP: Mechanical power
PEEP: Positive end-expiratory pressure
RR: Respiratory rate
